# If antibiotics aren’t always the answer—what is?

**DOI:** 10.1038/s41390-024-03632-3

**Published:** 2024-10-09

**Authors:** Damian Roland

**Affiliations:** 1 SAPPHIRE Group, Population Health Sciences, Leicester University, Leicester, UK; 2 Paediatric Emergency Medicine Leicester Academic (PEMLA) Group, Children’s Emergency Department, Leicester Royal Infirmary, Leicester, UK

The widespread implementation of Antibiotics has saved millions of lives and yet within 80 years of the introduction of penicillin, antimicrobial resistance has been associated with nearly 5 million deaths.^1^ It is postulated that AMR may overtake cancer as a leading cause of mortality by 2050^2^. It is obvious that antimicrobial stewardship is critical and yet health care clinicians in high income countries are faced with a persistent challenge of managing societal expectation for treatment and professional pressure on not missing treatable infection.

**Fig. 1:**
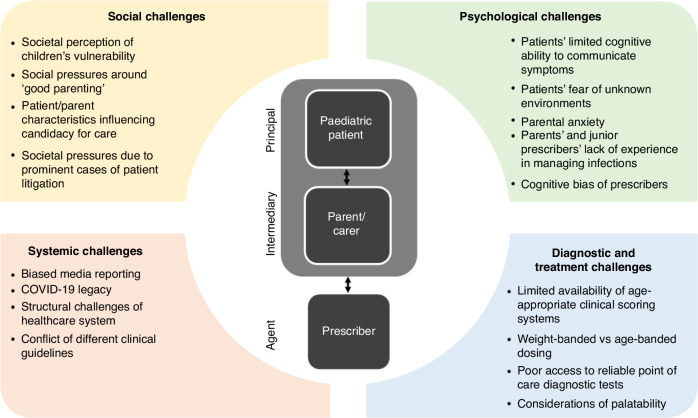
The challenges affecting the choices of stakeholders implicated in the decision process around the management of pediatric infections (From Krockow et al.^4^).

This tension is most apparent during acute childhood illness where the doctor or treating clinician (the agent) is providing a service to a patient (the principal). This principal-agent relationship^3^ is impacted (which may either be facilitating or complicating the interaction) by the presence of an intermediary such as a parent or carer (caregiver). The dynamics around the utilization of coherent antimicrobial stewardship principle for this triad are multi-factorial. Krockow et al.^4^ have used the concept of principal-agent relationships to highlight the different challenges which affect decision making when clinicians are managing pediatric infections (Fig. 1). This provides a framework for clinicians to reflect on prior to consultations by examining their own behaviors, i.e., what might their biases be, the caregivers’ motivations and the impact of the wider healthcare system on their treatment decision.

The psychological and social challenges on the principle-agent relationship are significant but the societal impact of antibiotic prescribing has been neatly encapsulated in a novel study by Poole et al.^5^. It provides some evidence that perhaps clinicians may be able to use with families to reduce anxiety and fear and provide a platform for a positivist approach as to why antibiotics might not be necessary. In their secondary analysis of data of a single-center, randomized controlled trial they investigated the association between antibiotic prescribing during an Emergency Department visit for Influenza and subsequent illness duration, but also were able to determine the impact on school absenteeism.

To note only (251/848) 30% of the original participants in the *Randomized Clinical Trial Assessing Point-of-Care Influenza and Other Respiratory Virus Diagnostics* (RAPID) trial, which evaluated the impact of rapid molecular testing of children with influenza on clinician decision making,^6^ were part of the secondary analysis, and there was significant survey follow up loss (238/848 28.1%). However the findings in this group (who were not admitted to hospital) are likely to be replicable; median age was 4.2 years, 52% were male, 40% white, 54% Hispanic, and 75% had government insurance. The study reported no statistically significant association with missed class days (incidence rate ratio [IRR]: 1.14 [0.86–1.50], *p* = 0.37) or days of illness (IRR: 1.06 [0.88–1.27], *p* = 0.55) for patients prescribed an antibiotic in the Emergency Department compared to patients who were not prescribed an antibiotic over the duration of illness.^5^

Their findings are consistent with other studies examining the impact of antibiotic prescriptions. In the CAP-IT study it was found that the lowest dose and shortest duration of antibiotic was effectively no different than longer doses and durations in the safe management of uncomplicated community acquired pneumonia.^7^ A Cochrane review from 2023 demonstrates in high income countries there is little or no benefit of prescribing antibiotics for otitis media,^8^ yet is some settings 80% of children receive them.^9^

Poole et al. highlight the parental pressure to prescribe antibiotics may be driven by the need to reduce time away from childcare (which prevents parents from working). They conclude that being able to reassure families that antibiotics do not alter time off nursery or school may aid antimicrobial stewardship. How much will this knowledge aid health care professionals in discussing the benefits and disadvantages of antibiotics with families? There are two significant challenges; the first is that caregivers will need to understand and accept that antibiotics don’t have the outcomes they think they may have. This will depend on the knowledge and experience of both the clinician and the caregiver with the challenges highlighted by Krockow et al. meaning that, even for the same child, different outcomes from consultations may result because of subtleties in societal pressures or clinicians’ moral duty.^10^ Secondly, and perhaps more importantly, even if the caregivers accept antibiotics are not answer what will address the concerns they have? There is no magic bullet for a child’s illness behaviors and the displacement of normal activities when unwell with a self-limiting illness. In high income countries societal norms push towards an expectation the child will get better quickly. Poole et al. show us that antibiotics are not the solution to having to time off work and school. But good antimicrobial stewardship may depend on us finding a solution or perhaps developing a stronger narrative that there never will be one.
